# Plasmonic enhancements of photoluminescence in hybrid Si nanostructures with Au fabricated by fully top-down lithography

**DOI:** 10.1186/1556-276X-7-629

**Published:** 2012-11-16

**Authors:** Koudo Nakaji, Hao Li, Takayuki Kiba, Makoto Igarashi, Seiji Samukawa, Akihiro Murayama

**Affiliations:** 1 Graduate School of Information Science and Technology, Hokkaido University, Kita 14, Nishi 9, Kita-ku, Sapporo, 060-0814, Japan; 2 Institute of Fluid Science, Tohoku University, 2-1-1 Katahira, Aoba-ku, Sendai, 980-8577, Japan; 3 Japan Science and Technology Agency, CREST, 5 Sanbancho, Chiyoda, Tokyo, 102-0075, Japan

**Keywords:** Au, Bio-nano-templates, Electron-beam lithography, Hybrid nanostructures, Neutral-beam etching, Plasmonic enhancements, Photoluminescence, Si nanodisk

## Abstract

The authors study plasmonic enhancements of photoluminescence (PL) in Si nanodisk (ND) arrays hybridized with nanostructures such as nanoplates of Au, where these hybrid nanostructures are fabricated by fully top-down lithography: neutral-beam etching using bio-nano-templates and high-resolution electron-beam lithography. The separation distance between the Si ND and Au nanostructure surfaces is precisely controlled by inserting a thin SiO_2_ layer with a thickness of 3 nm. We observe that PL intensities in the Si NDs are enhanced by factors up to 5 depending on the wavelength by integrating with the Au nanoplates. These enhancements also depend on the size and shape of the Au nanoplates.

## Background

Plasmonic effects induced by metallic nanostructures, such as remarkable enhancements of the intensity of photoluminescence (PL) in semiconductor nanostructures, are very attractive because optical responses of the semiconductor nanostructures can be controlled in the relatively wide spectral range [1-5]. However, fully top-down fabrication of semiconductor nanostructures exhibiting the plasmonic effects has not been established yet because the plasmonic effects appear only when optically active nanomaterials are close to the metallic nanostructures, typically at a distance of 10 nm. Therefore, conventional lithography techniques are not applicable to the fabrication of the plasmon-coupled hybrid nanostructures of semiconductors with metals.

To solve this problem, we have employed Si nanodisks (NDs) fabricated by damage-free neutral-beam etching using bio-nano-templates [6-8]. A closely packed two-dimensional alignment of the Si ND with a diameter of 10 nm and interspacing of 2 nm was formed, where the sheet density was 5 × 10^11^ cm^−2^. This alignment of the Si ND provides a good opportunity to prepare the abovementioned hybrid nanostructures, where metallic nanostructures can be closely arranged on a surface of the Si ND array. Moreover, we can place some NDs at the most appropriate position for realizing the plasmonic enhancement; this position must be very close to the edge of metallic nanostructures in the in-plane lateral direction as well as the perpendicular one. It is rather difficult to realize a similar situation when we use self-assembled quantum dots with sheet densities of 10^9^ to 10^10^ cm^2^. We demonstrate plasmonic enhancements of PL in a visible light region in the hybrid nanostructures of Si ND with Au. The separation distance between the Si ND layer and the Au nanostructure was precisely controlled by inserting a 3-nm-thick SiO_2_ layer. Improved optical functionalities such as efficient absorption and bright PL are highly expected for applications of the Si nanostructures to optical devices such as efficient solar cells and optical interconnections in Si-based integrated circuits.

## Methods

**Figure 1:**
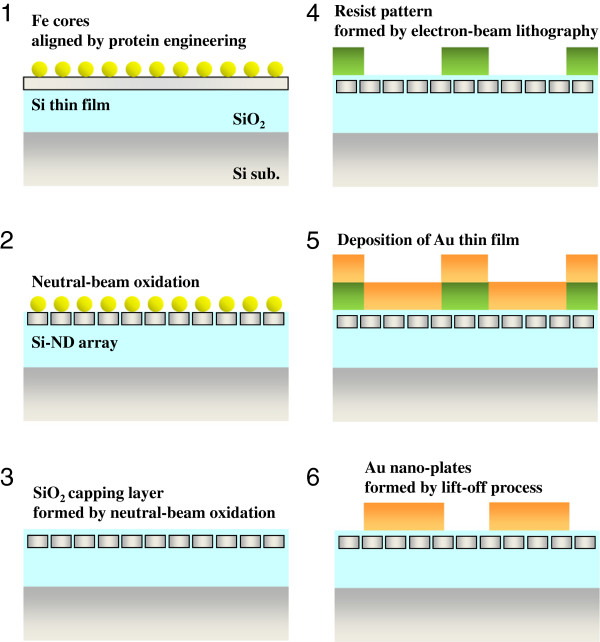
Fabrication processes of the hybrid nanostructure of Si NDs with Au nanoplates.

Si thin films with a thickness of 10 nm were deposited on surface-oxidized Si substrates, where a thickness of the oxidized SiO_2_ layer was 2 μm. High-density two-dimensional Si ND arrays were fabricated by neutral-beam etching using bio-nano-templates as an etching mask, consisting of ferritin supramolecules [6,7]. The diameter and interspacing of the Si NDs were intentionally designed at 10 and 2 nm, respectively, by protein engineering. After forming the Si ND array, 40-nm-thick Au thin films were deposited. Then, square-shaped Au patterns were fabricated by electron-beam lithography, where the length of one side of the Au square pattern was varied from 50 to 200 nm. Schematic illustrations of the fabrication processes of the hybrid Si NDs with Au nanoplates are shown in Figure 1. Neutral beams of oxygen radicals were irradiated from the top side of the two-dimensional alignment of Fe nanoparticles into the Si thin films, where the array of Fe particles was prepared by intentionally aligned protein supramolecules containing the Fe particles. This oxidation process formed the Si NDs surrounded by SiO_2_. After removing the Fe particles, top parts of the Si NDs were oxidized using the same neutral-beam oxidation process, and the resultant capping layer of SiO_2_ with a thickness of 3 nm was formed. A resist pattern was then formed by standard electron-beam lithography, and the Au thin film was deposited on the top of the resist pattern. Finally, the Au nanoplates were fabricated using a lift-off technique. Therefore, the SiO_2_ capping layer as well as the alignment of Si NDs was not affected by this lift-off process. The thickness variation of the oxide capping layer was estimated to be less than 10%.

PL spectra were observed at a temperature of 150 K using micro-PL equipment with a spatial resolution of 1 μm, corresponding to a diameter of an excitation light spot. An InGaN laser with a wavelength of 408 nm and an excitation power of 0.3 mW was used as an excitation light source.

## Results and discussion

**Figure 2:**
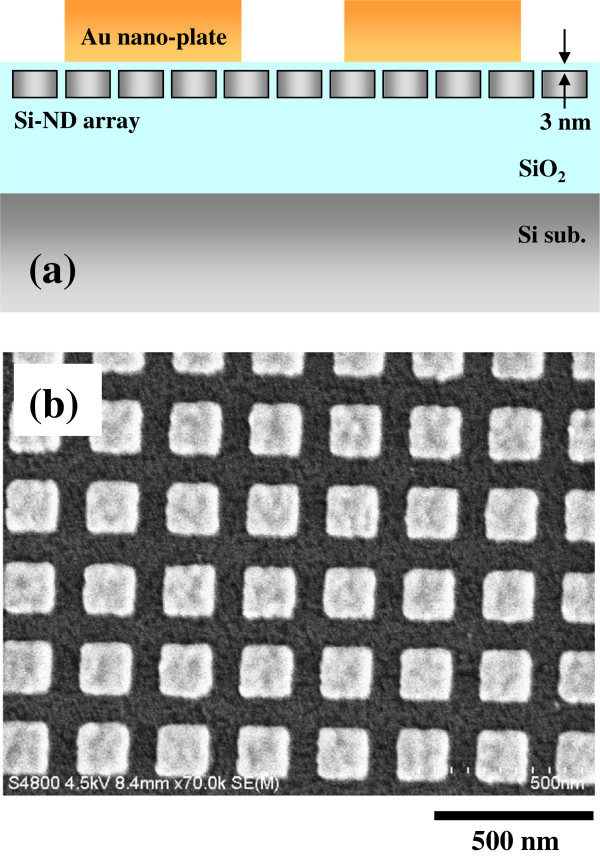
**Hybrid nanostructure and Au nanoplates.** (**a**) A schematic cross-sectional illustration of the hybrid nanostructure, where the Si ND array is separated from the Au nanoplates by a capping layer of SiO_2_ with a thickness of 3 nm. (**b**) A SEM image of the Au nanoplates fabricated on the Si ND array by electron-beam lithography. The length of one side of the Au square pattern was designed to be 150 nm.

Figure 2a shows a schematic cross-sectional illustration of the hybrid nanostructure of Si NDs with Au nanoplates, where the surfaces of the Si ND layer and Au nanoplates are separated by inserting a SiO_2_ layer with a thickness of 3 nm. The lateral diameter of the Si ND is 10 nm, and the minimum spacing among the NDs is 2 nm. An example of the square-shaped Au nanostructures fabricated on the top of the Si ND array is shown in Figure 2b. The length of one side of this Au square pattern is 150 nm.

**Figure 3:**
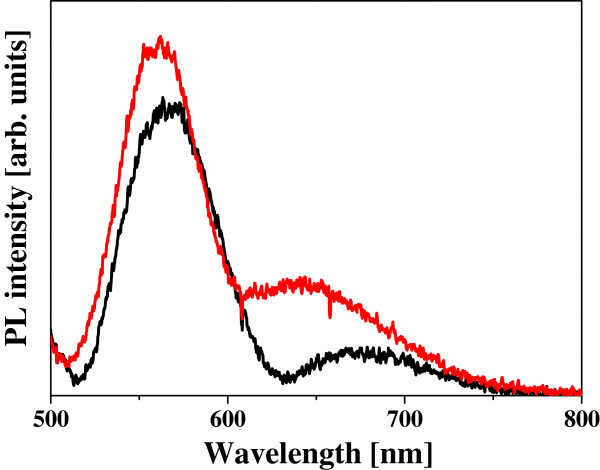
**Micro-PL spectra from Si ND arrays.** In the Si ND array hybridized with Au nanoplates, the length of one side of the Au plate is 200 nm (a red line). The Si ND array without the Au nanostructure (a black line).

**Figure 4:**
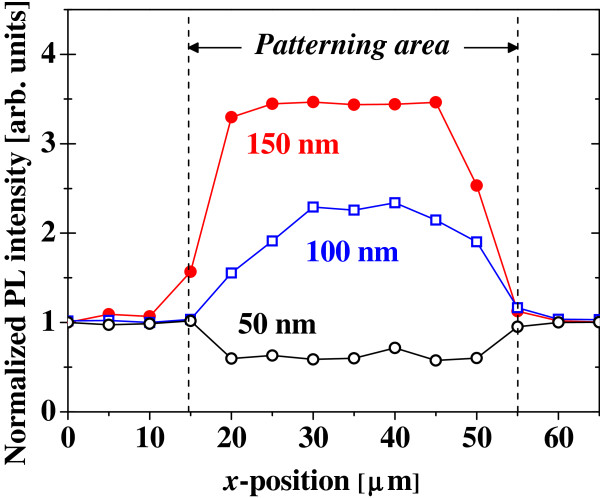
**PL intensities from the Si ND arrays.** The PL intensities with a wavelength of 625 to 675 nm in the Si ND arrays with various sizes of the Au nanoplate (a solid red circle, 150 nm; an open blue square, 100 nm; an open black circle, 50 nm) are plotted as a function of the lateral measurement position of the micro-PL. The PL intensity is normalized for that from the Si ND array at the position of *x* = 0 μm where the Au nanostructure is not formed. The patterning area of the Au nanostructure is indicated by broken lines (*x* = 15 to approximately 55 μm).

**Figure 5:**
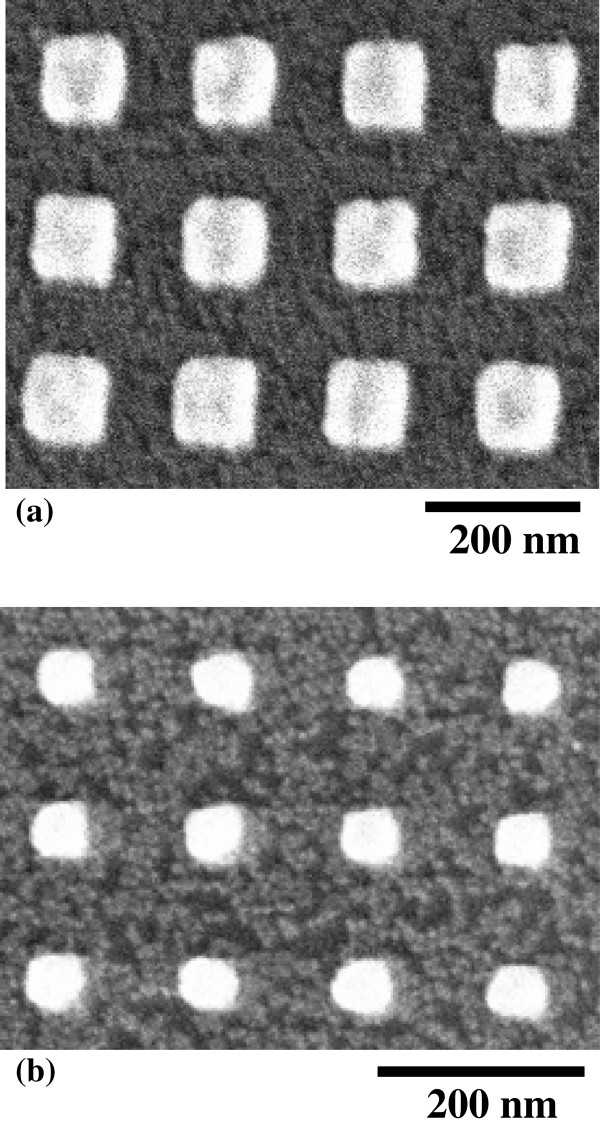
**SEM images of Au nanoplates fabricated on the Si-ND array by electron-beam lithography.** The designed lengths of one side of the Au square pattern are (**a**) 100 and (**b**) 50 nm, respectively.

Figure 3 shows the PL spectra from the Si ND arrays with and without the Au nanoplates. It is shown that the PL intensity of the Si NDs can be increased by integrating the Au nanoplates. The degree of the PL enhancement depends on the wavelength as can be seen, which will be discussed later. The PL intensities from the Si ND arrays with different sizes of Au nanoplates are plotted as a function of measurement position of the micro-PL in Figure 4. The PL intensity increases within the patterning area when the Au nanoplates with sizes of 150 and 100 nm are fabricated on top of the Si ND array. In contrast, the PL intensity in the case of Au nanoplates with a size of 50 nm decreases in comparison with that outside the patterning region. This is natural because the surface of the Si ND array is covered partly with the metallic nanostructures, and therefore, the incident light and PL are masked to some degrees. However, the PL intensities are markedly enhanced by the existence of Au nanoplates with larger sizes as mentioned above, although the surfaces of the Si NDs also are partly masked. We observe that PL intensities in the Si NDs are enhanced by factors up to 5 depending on the size and shape of the Au nanostructures as well as the wavelength. Therefore, we conclude that the Au nanoplates with lengths of 100 to 200 nm on one side show clear PL enhancements. The edge shape of these square patterns of Au has successfully been reproduced by electron-beam lithography, as shown in Figure 2b, while that of a 50-nm square pattern is not sharp because of the spatial resolution of our lithography. Scanning electron microscopy (SEM) images of the Au square patterns with smaller sizes, such as 100 and 50 nm, are shown in Figure 5. This fact strongly suggests that the PL intensities in the Si ND arrays are enhanced by plasmonic effects induced by the Au nanostructures because plasmon-enhanced local electric fields of lights are known to be highly sensitive to the edge shape of metallic nanostructures. We can arrange some Si NDs closely at the edge part of the Au nanoplates, which is important for inducing the plasmonic effects. Strong plasmonic effects are known to appear when optically active materials are placed at appropriate distances of 5 to 15 nm, depending on the shape of metallic nanostructures [9,10].

**Figure 6:**
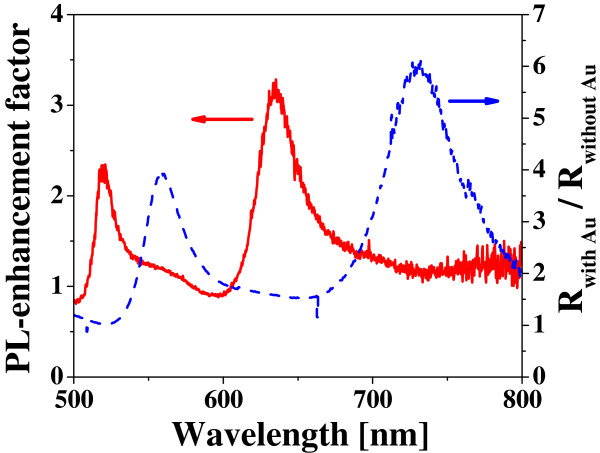
**An enhancement factor of the PL intensity.** The enhancement factor is plotted as a function of wavelength in the hybrid nanostructure of Si NDs with 150-nm square Au nanoplates (a solid red line), where the PL intensity of the Si NDs with the Au nanoplates is divided by that without the Au ones. The reflectivity change originating from the existence of Au nanoplates is also plotted as a function of wavelength (a broken blue line), where the reflectivity for the sample surface with the Au nanoplates is divided by that without the Au ones.

To understand the phenomena, we measured and compared reflectivity changes with the Au nanoplates on the surface of the Si ND array. We first measured a reflectivity spectrum for a bare surface of the Si ND array and then measured that for the Si ND array covered with the Au nanoplates. The reflectivity measured in the latter case with Au nanoplates was divided by the former one without Au. A typical result is shown in Figure 6, in comparison with the PL enhancement factor as a function of wavelength, where the PL intensity of the Si NDs with Au is divided by that without Au. As can be seen, the PL enhancements appear significantly when the reflectivity change becomes small. In a visible light region, lights are strongly reflected by the Au surface. Therefore, the reflectivity change caused by inserting a plane surface of Au should be large. The reflection of the bare Au surface actually starts to increase around 550 nm. The significantly smaller changes of the reflectivity, which are shown by a dip structure around 580 to 710 nm on the spectrum of reflectivity change, mean that incident lights are strongly absorbed, possibly originating from plasmon excitations in the Au nanostructures. The higher-order corrective interference, which can be generated from regular alignments of a large number of the metallic nanostructure, has not been observed because the incident and reflected lights are almost perpendicular to the film surface. In addition to this, the interference of the reflected lights between from a top surface of the Au nanoplates and a surface of the bottom Si substrate can be considered. However, the reflection spectra of the Au nanoplates, which were directly fabricated on the surface of Si substrates, were similar to those observed in the present hybrid Si NDs with the Au nanoplates. Therefore, we conclude that the dip structures observed on the reflection spectra can be attributed to effects of the nanostructured Au.

As can be seen, this spectral dip of the reflectivity change coincides well with the spectral peak at 640 nm of the PL enhancement. Therefore, the PL enhancements observed in these hybrid Si/Au nanostructures can be attributed to the excitations of localized surface plasmons around the Au nanoplates, particularly around sharp edges of the corner of the Au square pattern. The PL enhancement factors for the limited number of Si ND arranged at the appropriate places for strong plasmonic effects should be significantly higher than the averaged values observed by the present micro-PL, if we accept the above scenario.

## Conclusions

We have observed plasmonic enhancements of PL in hybrid nanostructures of Si NDs with Au nanoplates, where the Au nanoplate is separated from the surface of the Si ND array by inserting a SiO_2_ layer with a thickness of 3 nm. We demonstrate that PL intensities in the Si NDs are enhanced by integrating the Au nanoplates, depending on the wavelength. This enhancement also depends on the size and shape of the Au nanoplates. We find that the PL spectral region indicating the enhancement correlates well with the spectral dip of the reflectivity change originating from the Au nanoplates. Therefore, we attribute the PL enhancements observed in these hybrid Si/Au nanostructures to plasmonic effects induced by the square-shaped Au nanostructures.

## Abbreviations

ND: nanodisk; PL: photoluminescence; SEM: scanning electron microscopy.

## Competing interests

The authors declare that they have no competing interests.

## Authors′ contributions

KN fabricated the Au nanostructures. MI fabricated the Si NDs. KN, HL, TK, and AM performed the spectroscopic study and analyzed the structural and optical data. KN, TK, and AM drafted the manuscript. KN, TK, MI, SS, and AM conceived the fabrication process and participated in its design and coordination. All authors read and approved the final manuscript.
